# Correction: Cobalt-catalyzed, directed arylation of C–H bonds in *N*-aryl pyrazoles

**DOI:** 10.1039/d1ra90093e

**Published:** 2021-03-29

**Authors:** Tung T. Nguyen, Lam V. Le, Hai H. Pham, Dung H. Nguyen, Nam T. S. Phan, Ha V. Le, Anh N. Q. Phan

**Affiliations:** Faculty of Chemical Engineering, Ho Chi Minh City University of Technology (HCMUT) 268 Ly Thuong Kiet, District 10 Ho Chi Minh City Vietnam tungtn@hcmut.edu.vn pnqanh@hcmut.edu.vn; Vietnam National University Ho Chi Minh City Linh Trung Ward, Thu Duc District Ho Chi Minh City Vietnam

## Abstract

Correction for ‘Cobalt-catalyzed, directed arylation of C–H bonds in *N*-aryl pyrazoles’ by Tung T. Nguyen *et al.*, *RSC Adv.*, 2021, **11**, 9349–9352, DOI: 10.1039/D1RA00975C.

The authors regret that due to a typing error in the manuscript, the compound Ce(SO_4_)_2_ was written as CeSO_4_. All mentions of CeSO_4_ in the paper and ESI should be read as the commercially available cerium(iv) sulfate, Ce(SO_4_)_2_. The conclusions of the paper have not been affected.

The Royal Society of Chemistry apologises for these errors and any consequent inconvenience to authors and readers.

## Supplementary Material

